# Laparoscopic-assisted resection of a giant colonic diverticulum: a case report

**DOI:** 10.1186/1752-1947-3-7075

**Published:** 2009-05-28

**Authors:** Jacqueline E Collin, Gurprit SS Atwal, William K Dunn, Austin G Acheson

**Affiliations:** 1 Department of Colorectal Surgery, Queens Medical Centre, Nottingham Universities NHS Trust Nottingham NG7 2UH UK; 2 Department of Histopathology, Queens Medical Centre, Nottingham Universities NHS Trust Nottingham NG7 2UH UK; 3 Department of Radiology, Queens Medical Centre, Nottingham Universities NHS Trust Nottingham NG7 2UH UK

## Abstract

**Introduction:**

Diverticular disease of the colon is a common benign condition. The majority of patients with diverticular disease are asymptomatic and are managed non-operatively, however complications such as perforation, bleeding, fistulation and stricture formation can necessitate surgical intervention. A giant colonic diverticulum is defined as a diverticulum larger than 4 cm in diameter. Despite the increasing incidence of colonic diverticular disease, giant colonic diverticula remain a rare clinical entity.

**Case presentation:**

This is the first reported case of laparoscopic-assisted resection of a giant colonic diverticulum. We discuss the symptoms and signs of this rare complication of diverticular disease and suggest investigations and management. Reflecting on this case and those reported in the literature to date, we highlight potential diagnostic difficulties and consider the differential diagnosis of intra-abdominal gas-filled cysts.

**Conclusion:**

The presence of a giant colonic diverticulum carries substantial risk of complications. Diagnosis is based on history and examination supported by abdominal X-ray and computed tomography findings. In view of the chronic course of symptoms and potential for complications, elective surgical removal is recommended. Colonic resection is the treatment of choice for this condition and, where possible, should be performed laparoscopically.

## Introduction

Diverticular disease of the colon is a common benign condition that occurs in excess of 60% in those aged over 70 years [1,2]. It is generally a disease of the western world and the incidence appears to be increasing [3,4]. The majority of patients with diverticular disease have involvement of the sigmoid colon. These patients are frequently asymptomatic, when the condition is known as diverticulosis, and the diagnosis is made incidentally. Diverticular disease refers to symptomatic diverticula; patients commonly present with bloating, abdominal pain, flatus and rectal bleeding. Inflammation of diverticula, known as diverticulitis, classically causes left-sided abdominal pain, change in bowel habit with passage of mucous or fresh blood, and systemic upset.

About 5% of patients who have symptomatic diverticula experience complications such as perforation, bleeding, fistulation and stricture formation which can necessitate surgical intervention.

A giant colonic diverticulum (GCD) is defined as a diverticulum larger than 4 cm in diameter [4]. Some as large as 40 cm have been reported in the literature [5]. The mean age of presentation of GCD mirrors that of diverticular disease with the majority presenting after the sixth decade [1,4].

The presentation of GCD is variable, ranging from the asymptomatic patient (4%) to a host of non-specific gastrointestinal (GI) symptoms with only 10% of patients presenting with an abdominal mass [4]. GCD carries a substantial risk of complications and elective surgical removal is recommended [6].

Despite the increasing incidence of colonic diverticular disease, GCD remains a rare clinical entity [7]. We report a case of a 53-year-old man who underwent a laparoscopic-assisted sigmoid colectomy for treatment of a symptomatic giant diverticulum. This is the first reported case of laparoscopic-assisted resection of a GCD.

## Case presentation

A 53-year-old white Italian man initially presented to gastroenterologists with a 5-week history of dyspepsia, epigastric pain and a palpable mass in the left hypochrondrium. There was no history of anorexia, dysphagia, weight loss, change in bowel habit or gastrointestinal blood loss. His past medical history included early Alzheimer’s disease and discoid lupus.

Examination revealed a well circumscribed, mobile mass in the left hypochrondrium extending above the level of the ribs raising the possibility of an enlarged spleen. There was no palpable lymphadenopathy.

A blood film showed atypical myelomonocytic cells but a subsequent bone marrow aspiration was normal. All other routine blood tests were within normal limits. An abdominal ultrasound scan demonstrated a normal spleen and a separate gas-filled cyst in the left hypochondrium.

Over the next few weeks, the patient developed diarrhoea and lost 3 kg in weight. He reported that the mass appeared to be fluctuating in size.

**Figure 1.**
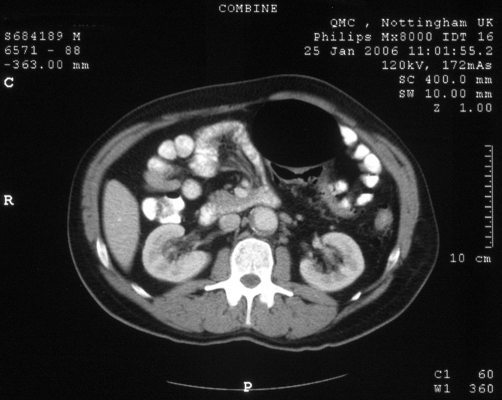
Abdominal computed tomography demonstrating a large gas-filled structure in the left upper abdomen arising from the sigmoid colon.

An abdomen computed tomography (CT) scan (Figure 1) demonstrated a large gas-filled structure measuring 11 cm × 12 cm, appearing to arise from the sigmoid colon, displacing the adjacent small and large bowel loops. The features were consistent with a giant sigmoid diverticulum.

**Figure 2.**
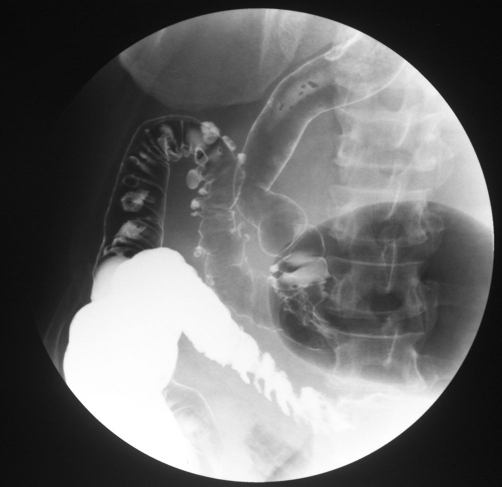
Barium enema: the air filled cavity did not fill with barium nor did it change in size on insufflation.

He was referred to colorectal surgeons and a barium enema was performed to further assess the extent of the diverticular disease. This confirmed moderate sigmoid diverticulosis but did not demonstrate direct communication between the colon and the giant cyst (Figure 2). The diagnosis of GCD was discussed with the patient and definitive surgical management was advised. Initially, the patient was reluctant to have surgery, but over the next 6 months, he experienced two further episodes of acute abdominal pain necessitating hospital admission. Both episodes were similar in nature with pain as the predominant symptom; an abdominal X-ray (AXR) taken on admission demonstrated the gas-filled structure and in the absence of raised inflammatory markers, a normal white cell count and no fever, the diagnosis of enlarging GCD was made. Both episodes settled quickly with bowel rest and intravenous fluids. The patient then agreed to surgical intervention.

**Figure 3.**
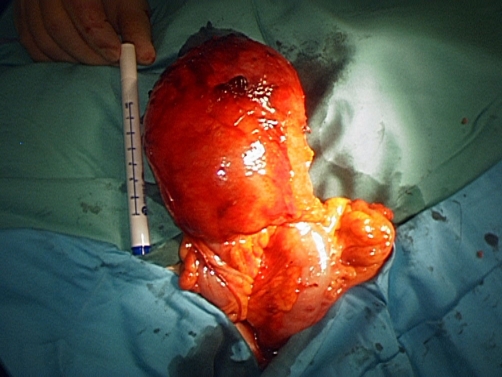
Externalised sigmoid colon and anti-mesenteric giant cyst.

A laparoscopic-assisted sigmoid colectomy was performed 6 weeks later. Four 12 mm ports were inserted and pneumoperitoneum achieved. Three of the ports were positioned along the lateral edge of the right rectus abdominus muscle and triangulated to provide optimum access to the left colon. The fourth port was in the left iliac fossa. The large cystic structure was clearly visible in the left hypochondrium at the apex of a long mobile loop of sigmoid colon on the anti-mesenteric border (Figure 3). The remaining sigmoid colon had macroscopic evidence of mild diverticulosis. The diverticulum was attached to the lateral abdominal wall adjacent to the spleen by adhesions. These adhesions were divided laparoscopically by a combination of scissor diathermy and ultracision. The sigmoid colon was then fully mobilised from lateral to medial but no attempt was made to divide the mesenteric vessels intracorporeally in view of the fact this was benign disease and the sigmoid was long and tortuous. The mobilised sigmoid colon was externalised through a 7 cm incision in the left iliac fossa. A wound protector was used during extraction of the cyst and a decision was made not to decompress it before removal in order to keep possible contamination down to a minimum. The sigmoid colon was resected along with the diverticulum and a hand-sewn primary anastomosis was performed extracorporeally.

The patient made an excellent postoperative recovery and was discharged on the fourth postoperative day.

Macroscopic assessment of the segmental colonic resection confirmed the presence of diverticular disease with an associated giant cyst measuring 11 cm in maximal diameter. The wall of the cyst measured 0.6 to 1 cm in thickness. Microscopically, it did not contain any elements of bowel wall and instead was composed of reactive scar tissue with foreign body type giant cell reaction. The presence of plant material admixed with inflammatory debris was thought to be indicative of faecal matter and suggested a direct communication between the cyst and bowel lumen. However, this was not identified histologically. There was no evidence of dysplasia or malignancy. In accordance with the classification suggested by Steenvoorde *et al.* [4], the histological features were consistent with Type II GCD (Table 1).

**Table 1.** Histological classification of giant colonic diverticulum; from Steevoorde *et al.* [4]

Type	Name	Aetiology	Histology
I	Pseudo-diverticulum	Unidirectional ball-valve mechanism	Remnants muscularis mucosa/muscularis propria
		Gas producing organism	
II	Inflammatory	Local perforation of mucosa with abscess cavity	Reactive scar tissue, no bowel tissue
III	True diverticulum	Congenital	All three layers of bowel tissue, communicating with gut lumen

## Discussion

Diverticular disease of the colon is a significant cause of morbidity and mortality in the western world and its frequency increased throughout the whole of the 20^th^ century [3,8]. Since it is a disease of the elderly, and with an ageing population, it can be expected to occupy an increasing portion of the surgical and gastroenterological workload [3,8].

GCDs are defined as those that are larger than 4 cm in diameter [4,5] and with the increasing incidence of diverticular disease [3,8], it is likely that the incidence of these giant lesions will increase further. Awareness of the presenting symptoms, investigations, differential diagnosis and management is therefore important.

As in our patient, it is not unusual for these patients to undergo multiple investigations before making the correct diagnosis. Plain supine abdominal X-ray is the simplest and most readily available investigation and should be used as the first line in suspected cases. If a large air filled structure with or without fluid levels is visualised then an abdominal CT scan would be indicated. Barium enema failed to demonstrate a communication between the giant diverticulum and the colon in approximately one-third of reported cases [1,4]. It is therefore not surprising that no communication was identified in our patient. Barium enema can be useful at providing valuable information regarding the extent of further diverticula.

The use of abdominal ultrasound has been reported to be helpful in only 25% of cases [4]. Early colonoscopy is advised in the setting of persistent or frequent acute diverticulitis to rule out concurrent pathology [9]. Our patient was admitted acutely on two occasions a few months apart, however, the symptoms and signs were not suggestive of acute diverticulitis but were felt to be in keeping with enlarging GCD therefore colonoscopy was not performed.

The role of colonoscopy in diagnosing GCD is limited. The ostium between the diverticulum and the colon is frequently too small to be detected [1,2,4] and even in cases with wide necked GCD, the ostium is not detected on sigmoidoscopy [1]. The combination of a large soft, mobile mass in an elderly patient and a lucent cystic structure related to the sigmoid colon on AXR should suggest the diagnosis of a GCD [6].

Other causes for intra-abdominal gas-filled cysts, radiologically mimicking GCD [2], along with their principal distinguishing features, are summarised in Table 2. Steenvoorde *et al.* suggested a histological classification of GCD based on three subtypes (Table 1). The distinction between type I and II has not always been made with both categories being discussed as one entity in many papers [5]. Theories behind the formation of GCD type I and II are speculative and not mutually exclusive. The suggested aetiology of type I is based on the premise that the communication between the GCD and the colon is small enough to preclude the escape of air from the diverticulum [1]. The two most widely accepted theories are, a unidirectional ball-valve mechanism causing gas entrapment and infection with gas producing organisms leading to progressive diverticula enlargement [5]. However, such theories do not convincingly explain the existence of type I GCD with wide necks.

**Table 2.** Differential diagnosis of intra-abdominal gas-filled cysts

Condition	Age at presentation (years)	Diagnostic investigation	Distinguishing features
GCD	>60	AXR, CT	>4 cm in size, air filled cyst
			Usually arises from the sigmoid colon
			Anti-mesenteric border [2]
			Associated diverticular disease
			60% palpable abdominal mass [4-6]
Pneumatosis cystoides	30-50 [11]	CT	Usually asymptomatic
			Symptoms: abdominal distension, discomfort, mucoid stools
			15% primary/idiopathic
			85% secondary: IBD, diverticulosis, pulmonary disease
			Numerous small pockets within bowel wall
			Affects small and large bowel [11]
Meckels diverticulum	<30	Tech^99^, CT	2% population, 95% asymptomatic
			<2 cm in length
			PR bleeding most common presenting symptom in children
			Other symptoms: abdominal obstruction, inflammation, intussusception, ulceration and perforation
			Contain all layers of bowel wall
			Anti-mesenteric border, within 100 cm of ileocaecal valve
Volvulus (caecal/sigmoid)	>70	AXR, Sigmoidoscopy	Associated bowel obstruction
			Redundant sigmoid colon, past history of chronic constipation
			Haustra visible on distended loop on AXR [12]
Duplication cysts	<2	CT, USS, AXR	Anywhere along GI tract, most common in ileum
			Can be single/multiple
			50% have associated anomalies
			Wide range of symptoms pending location
			Mesenteric side, elongated in shape
			90% Non-communicating with gut lumen
			All bowel layers [12]
Emphysematous cystitis	>40	AXR, CT, USS	Due to bacterial fermentation of urinary glucose
			Gas production in bladder lumen and wall
			Assoc with diabetes, neurogenic bladder, bladder outlet obstruction, recurrent urinary tract infections
			Symptoms include dysuria, frequency, pneumaturia
			Distended tympanic mass arising from pelvis
			Most commonly due to *Escherichia coli*
Emphysematous cholecystitis [12]	>40	AXR, CT	RUQ pain, vomiting, pyrexia +/- RUQ mass
			Increased risk with diabetes and gallstones
			Infection usually due to *Clostridium perfringes*
			More risk of gangrene and perforation than with acute cholecystitis
Intra-abdominal abscess	-	CT	Source of intra-abdominal sepsis
			Swinging pyrexia
			Palpable mass

Type II is postulated to form following a subserosal perforation resulting in a walled off abscess cavity that gradually enlarges to giant size [7]. Type III contains all layers of bowel wall and structurally resembles a duplication cyst [7] but is in continuity with the gut lumen and occurs in adults. Approximately 20% of GCD show no evidence of a communicating ostium between the colon and the diverticulum and it is thought that this tract may be lost due to inflammatory changes [5].

Surgical management of a GCD involves either removing the diverticulum in isolation or colectomy. Diverticulectomy is not recommended as the mouth of the diverticulum may be wide and the surrounding inflammation could increase the potential for breakdown of the colonic closure [2]. Giant diverticula appear mostly (81%) in the sigmoid colon [5] with 50% of patients having concurrent sigmoid diverticula [4], thus sigmoid colectomy with primary end-end anastomosis [7] is the preferred operation. Resection is frequently difficult due to the inflammatory diverticulum and it is often densely adherent to surrounding structures [2]. In complicated or emergency cases, the safest surgical solution may be a Hartmann’s procedure [7].

The advent of laparoscopic colorectal surgery has had a significant impact on the postoperative recovery period for patients undergoing surgical resections for both benign and malignant colorectal disease. The most important advantages to the patient of laparoscopic surgery are reduction in pain, more rapid recovery of bowel function, better cosmetic results and a shorter hospital stay [5,6,10]. Our patient was fit for discharge on day four and this undoubtedly was due to the minimally invasive surgery performed. Based on our experience in this patient along with the recommendations of the Cochrane review group [10], surgical removal a GCD should be minimally invasive using laparoscopic techniques.

## Conclusion

Giant colonic diverticulum is a rare entity that is associated with a significant complication rate. The presentation of GCD is variable ranging from the asymptomatic patient (4%) to a host of gastrointestinal symptoms including abdominal pain (68%), constipation (18%), rectal bleeding (13%), vomiting (12%), abdominal distension (11%), diarrhoea (11%) and abdominal mass (10%) [4]. Accurate diagnosis, although difficult, can be achieved using a combination of clinical examination, plain AXR and CT scanning.

The presence of a GCD carries substantial risk of complications (12% to 19%) including inflammation, perforation, abscess formation, fistula formation, urinary obstruction [7], volvulus, small bowel obstruction and rarely, the development of adenocarcinoma [1,4].

In view of the chronic course of symptoms and potential for complications, elective surgical removal is recommended [6]. Colonic resection is the treatment of choice for this condition and, where possible, should be performed laparoscopically.

## Abbreviations

AXR: abdominal X-ray
CT: computerised tomography
GCD: giant colonic diverticulum
GI: gastrointestinal
IBD: inflammatory bowel disease
PR: per rectum
RUQ: right upper quadrant
Tech^99^: technetium-99
USS: ultrasound scan
